# Feeding Uninvited Guests: mTOR and AMPK Set the Table for Intracellular Pathogens

**DOI:** 10.1371/journal.ppat.1003552

**Published:** 2013-10-03

**Authors:** Jason Brunton, Shaun Steele, Benjamin Ziehr, Nathaniel Moorman, Thomas Kawula

**Affiliations:** Department of Microbiology and Immunology, University of North Carolina at Chapel Hill, Chapel Hill, North Carolina, United States of America; Columbia University, United States of America

Most pathogenesis studies focus on pathogen virulence attributes that mediate host colonization, toxicity, or immune evasion. Some studies focus on how pathogens employ active mechanisms to acquire essential nutrients such as iron and vitamins from the host by producing siderophores or avidins. In order to prevent pathogen nutrient acquisition, host cells employ a process called nutritional immunity to sequester these nutrients, particularly iron, from invading pathogens [1]. However, relatively little attention has been paid to understanding the mechanisms by which pathogens parasitize energy and catabolic substrates from the host even though several host and pathogen metabolic genes, including those in central carbon metabolism, are regularly identified as required for growth in the host [2], [3]. This issue is particularly important for intracellular pathogens that must compete with the host cell for energy and nutrient sources.

How and where do intracellular pathogens obtain sufficient amounts of energy and nutrients to support their replication? Pathogens may either parasitize existing energy stores or manipulate the host cell to create usable energy and anabolic precursor metabolites. Several recent studies have identified the host AMP-activated protein kinase (AMPK) and mammalian target of rapamycin (mTOR) kinases as two important regulators of cellular metabolism whose activities are often altered during infection. However, the AMPK/mTOR pathway also regulates autophagy, which can destroy cytosolic pathogens. While the evasion of autophagy by pathogens is well appreciated, recent work suggests that both the AMPK/mTOR pathway and autophagy itself can provide intracellular metabolites that support intracellular pathogen replication.

## AMPK and mTOR Regulate Energy Homeostasis

**Figure 1:**
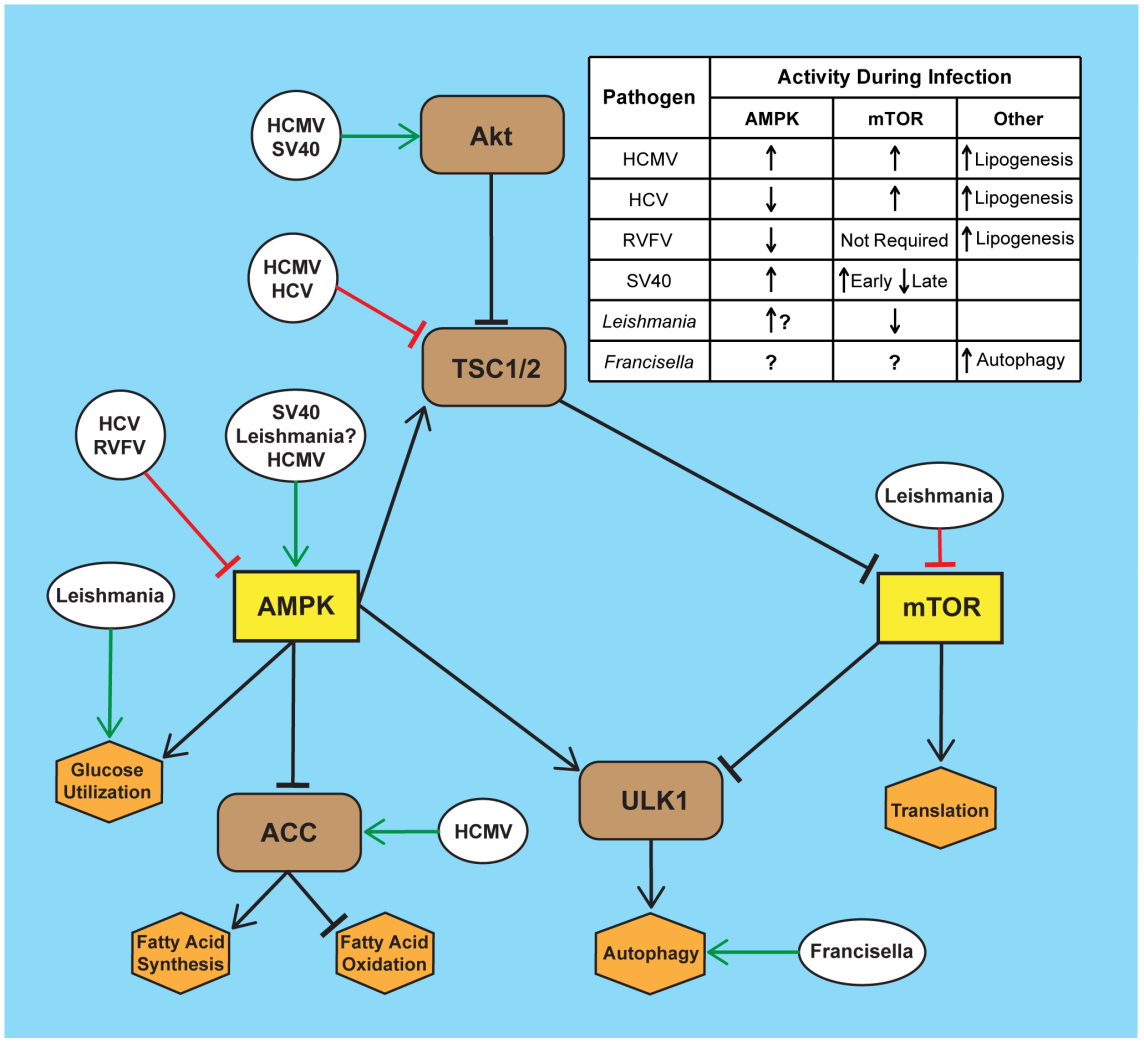
Infection by diverse pathogens impacts AMPK and mTOR signaling. Several intracellular pathogens manipulate the AMPK/mTOR pathway during infection through either directly targeting AMPK or mTOR or by targeting the upstream or downstream pathways. Depicted here are specific points of manipulation in the mTOR/AMPK pathway by human cytomegalovirus (HCMV), hepatitis C virus (HCV), Rift Valley fever virus (RVFV), simian virus 40 (SV40), *Leishmania*, and *Francisella* species. The table summarizes the resulting effects on the activities of mTOR and AMPK from infection by the specific pathogen.

During times of limited nutrient availability, intracellular ATP levels fall, with a corresponding increase in AMP levels. Within eukaryotic cells the increased AMP∶ATP ratio induces AMPK activity, which in turn initiates a series of signaling events that stimulate energy and nutrient acquisition [4]. For example, activated AMPK stimulates glycolytic flux, increases glucose uptake, and induces fatty acid oxidation (Figure 1). Together these events allow the cell to use its existing metabolic stores and also acquire new sources of energy. At the same time, activated AMPK limits energy consuming processes. Activated AMPK conserves energy by globally reducing protein synthesis, which perhaps is the most energy-intensive process in eukaryotic cells. AMPK limits protein synthesis by antagonizing the mTOR kinase, and mTOR kinase activity is necessary for formation of the elF4F complex, which is critical for translation initiation. In addition, mTOR and AMPK inversely regulate the recycling of existing intracellular metabolites through their effects on autophagy. Active AMPK stimulates autophagic breakdown of macromolecular complexes in the cell, thus producing energy and nutrients. In contrast, active mTOR suppresses autophagy to promote cell growth and proliferation. In a simplified view, when energy is low AMPK is active and mTOR is inhibited. This stimulates energy-producing processes and inhibits energy consumption thereby providing sufficient energy to support cell viability. Although AMPK and mTOR have additional roles outside of cellular metabolism, here we focus on the effects of AMPK and mTOR on cellular metabolism during infection by intracellular pathogens.

## Manipulation of Both AMPK and mTOR by Intracellular Pathogens

In order to achieve optimal levels of proliferation, many pathogens must manipulate activity of AMPK and mTOR. Interestingly, several viral pathogens have evolved strategies that allow for the induction of both AMPK and mTOR activity. For example, infection with human cytomegalovirus (HCMV) increases both AMPK and mTOR activity [5]. To acquire sufficient energy for viral growth, HCMV infection increases glycolytic flux in an AMPK-dependent manner [2], [6]. However, HCMV must strictly regulate AMPK activity during infection, as treatment of infected cells with chemicals that strongly activate or inhibit AMPK can limit viral replication [6], [7]. Interestingly, HCMV replication also requires fatty acid synthesis, which should be inhibited when AMPK is activated. Yet fatty acid synthesis is maintained during HCMV infection through a mechanism that requires mTOR activation [8]. How does HCMV allow for the activation of both AMPK and mTOR? The answer lies in part in the activity of the HCMV UL38 protein (pUL38). pUL38 binds and inhibits the TSC1/2 complex, which is necessary for antagonism of mTOR by activated AMPK [9]. HCMV thus uncouples AMPK/mTOR signaling resulting in increased energy production and lipid synthesis, both of which contribute to virus replication.

Simian virus 40 (SV40) infection also stimulates both AMPK and mTOR activity. SV40 small T antigen is both necessary and sufficient for AMPK activation [10], [11]. This function of small T antigen may provide critical nutrients needed for viral replication. mTOR activity is induced early in infection but inhibited as infection progresses. The mechanism driving the early induction of mTOR activity is unknown, but may be the result of Akt activation by the SV40 T antigens. However, the inhibition of mTOR activity during the late stage of infection is due to the effects of the SV40 small T antigen [11]. While activated AMPK would seemingly reduce SV40 protein synthesis, the expression of SV40 late proteins is driven by an internal ribosome entry site (IRES) that allows for efficient late mRNA translation when mTOR is inhibited [12]. It is likely that other pathogens employ active mechanisms to balance AMPK and mTOR signaling to allow for both catabolic and anabolic processes essential for pathogen replication, similar to HCMV and SV40.

## Inhibiting AMPK or Inducing mTOR Can Provide Essential Substrates for Pathogen Replication

Enveloped viruses require host lipids to generate the virion membrane. Activated mTOR stimulates fatty acid and lipid synthesis, and therefore could prove beneficial for virus assembly. In fact, host lipid metabolism is essential for the hepatitis C virus (HCV) life cycle and is highly regulated during infection [13], [14]. HCV infection limits AMPK activity and chemical induction of AMPK suppresses viral replication and inhibits fatty acid synthesis in HCV-infected cells [15]. Consistent with AMPK suppression, mTOR activity is increased during HCV infection through increased Akt signaling and decreased TSC1/2 expression [16]. However, this raises the question of how HCV acquires significant energy sources for viral replication in an AMPK-inhibited, mTOR-activated metabolic state? The answer may be the temporal regulation of host signaling and nutrient usage. Glucose import is required for viral replication and glycolytic flux is induced early during HCV infection [14], [17]. The products of glycolysis are likely diverted to fatty acid synthesis, as TCA flux and oxidative phosphorylation are reduced in HCV-infected cells [14], [18]. Later during infection, glucose uptake is reduced, while β-oxidation and amino acid catabolism are increased [14]. It is therefore possible that HCV temporally regulates AMPK and mTOR activity to achieve significant viral protein translation and lipid production, yet still obtain sufficient energy to support virus replication. Some bacterial pathogens may benefit from inhibiting AMPK and activating mTOR by inducing lipid synthesis, as *Mycobacterium tuberculosis* and *Chlamydia trachomatis* utilize fatty acids derived from lipid droplets [19], [20]. However, it is unknown how these bacteria affect host metabolic signaling to acquire nutrients.

AMPK activation also inhibits the replication of several arboviruses, including Rift Valley fever virus (RVFV) [21]. RVFV replication can be rescued in the presence of activated AMPK by providing cells with excess palmitate [21]. This suggests that AMPK inhibition is required to provide lipids essential for viral replication. The HIV-1 Tat protein inhibits the host SIRT1 protein resulting in AMPK inhibition [22]. Interestingly, AMPK induction inhibits lytic HIV replication, but is involved in reactivation of latent HIV genomes suggesting that AMPK activity may have different roles in acute and persistent infection [23].

## AMPK Activation May Benefit Replication of Diverse Pathogens

It takes a lot of energy to make hundreds, thousands, or potentially millions of new parasites, bacteria, or viruses. It seems logical that intracellular pathogens that undergo significant intracellular growth would activate AMPK due to the energetic demands placed on the infected cell. Activation of AMPK could provide several benefits for intracellular pathogens. The increased glucose uptake, glycolysis, and fatty acid breakdown would increase available intracellular energy and nutrient pools needed for pathogen replication. For example, *Leishmania donovani* amastigotes (the parasitic form that grows inside macrophages) preferentially generate energy through fatty acid oxidation and amino acid catabolism [24], suggesting *L. donovani* acquires fatty acids and amino acids from the infected host cell. Consistent with this finding, transcriptomic analysis of macrophages infected with the related parasite *Leishmania major* suggests that infected cells increase glucose transport, glycolysis, and starch degradation [25]. While it is currently unknown how *Leishmania* alters host metabolic processes, a reasonable hypothesis is that intracellular *Leishmania* activates AMPK to benefit parasite replication. Activated AMPK could stimulate increased glucose utilization and autophagy, thus creating elevated levels of anabolic precursor pools for parasite growth. Parasite replication requires the *Leishmania* protein GP63, which cleaves and inactivates mTOR to reduce type I interferon production, thus AMPK activation could further benefit parasite replication by inhibiting mTOR [26]. Viral pathogens may also benefit from AMPK activation. Measles virus requires β-oxidation for replication [27], but it is unknown if the virus manipulates AMPK for energy generation. It would be interesting to determine if these intracellular pathogens and others induce AMPK to generate energy and nutrients for growth.

## Autophagy Provides Intracellular Pathogens with Nutrients

Autophagy is an essential cellular process that recycles cellular constituents from macromolecular complexes under conditions of nutrient stress. As discussed above, autophagy is positively regulated by AMPK and negatively regulated by mTOR. However, autophagy also functions as a host defense mechanism that destroys intracellular pathogens through a process termed xenophagy. While generally viewed as detrimental for intracellular pathogens, some bacteria and viruses use autophagosomes as a replicative niche [28]. Whether these pathogens benefit or simply tolerate residing in autophagosomes remains unclear. However, it may be that replicating in a site where free nutrients are accumulating provides pathogens with a competitive edge for the acquisition of nutrients. This concept is supported by recent evidence that intracellular pathogens may use autophagy to acquire energy and nutrients for growth. Dengue virus–induced autophagy degrades lipid droplets. This increases free fatty acids levels in the cell and stimulates β-oxidation, which is required for efficient dengue virus replication [29]. Similarly, we have found that *Francisella tularensis* growth is impaired in autophagy-deficient host cells. Bacterial growth was restored in autophagy-deficient cells by supplying the infected cells with excess pyruvate or amino acids. Since *F. tularensis* replicates within the cytosol of host cells, our results suggest that intracellular *F. tularensis* uses autophagy to increase cytosolic nutrient pools that support bacterial growth [30]. Interestingly, *F. tularensis* avoids engulfment by classical autophagosomes [31] and instead induces an alternative form of autophagy that is required for bacterial replication [30]. It is attractive to speculate that other intracellular pathogens manipulate autophagy to avoid xenophagic destruction, while simultaneously benefiting from autophagy-derived nutrients.

## Conclusion

AMPK and mTOR are critical regulators of host cell metabolism making them logical targets for manipulation by invading pathogens. The energetic burden of the host cell to create hundreds or more pathogens should deplete cellular ATP levels, thus activating AMPK. AMPK induction stimulates host processes to produce energy and nutrients that the pathogen could then steal from the host. This idea suggests AMPK activation may be a common theme among infection by successful intracellular pathogens. On the other hand, mTOR signaling stimulates protein and lipid synthesis, which could be beneficial for many viral pathogens; whereas mTOR modulation is likely less important for free-living bacteria pathogens and parasites that supply their own biosynthetic and translation machinery. Identifying what nutrient sources are required for intracellular growth and how host metabolic signaling is manipulated by infection is being investigated in viral pathogenesis, yet remains poorly understood in bacterial and parasitic pathogenesis.

Manipulating host metabolism is an attractive approach to controlling infection as targeting the host rather than the pathogen should considerably reduce the ability of pathogens to develop drug resistance. Several drugs already in clinical use target the AMPK or mTOR kinases to treat diseases such as cancer and diabetes. The studies described above suggest that these drugs may have additional uses in treating infections with intracellular pathogens. As our understanding of pathogen manipulation of host metabolism grows, it may also be possible to develop inhibitors of specific host metabolic pathways hijacked by intracellular pathogens. Identifying the essential nutrients required for intracellular pathogen proliferation and the host pathways manipulated to acquire these nutrients will be a significant step in understanding the requirements for viral, bacterial, and parasitic pathogenesis and identifying new targets for novel therapeutics.

## References

[1] Hood MI, Skaar EP (2012) Nutritional immunity: transition metals at the pathogen-host interface. Nat Rev Microbiol 10: 525–537. 22796883 10.1038/nrmicro2836 PMC3875331

[2] Terry LJ, Vastag L, Rabinowitz JD, Shenk T (2012) Human kinome profiling identifies a requirement for AMP-activated protein kinase during human cytomegalovirus infection. Proc Natl Acad Sci U S A 109: 3071–3076. 22315427 10.1073/pnas.1200494109 PMC3286917

[3] Pechous RD, McCarthy TR, Zahrt TC (2009) Working toward the future: insights into Francisella tularensis pathogenesis and vaccine development. Microbiol Mol Biol Rev 73: 684–711. 19946137 10.1128/MMBR.00028-09 PMC2786580

[4] Inoki K, Kim J, Guan KL (2012) AMPK and mTOR in cellular energy homeostasis and drug targets. Annu Rev Pharmacol Toxicol 52: 381–400. 22017684 10.1146/annurev-pharmtox-010611-134537

[5] Walsh D, Perez C, Notary J, Mohr I (2005) Regulation of the translation initiation factor eIF4F by multiple mechanisms in human cytomegalovirus-infected cells. J Virol 79: 8057–8064. 15956551 10.1128/JVI.79.13.8057-8064.2005 PMC1143722

[6] McArdle J, Moorman NJ, Munger J (2012) HCMV targets the metabolic stress response through activation of AMPK whose activity is important for viral replication. PLoS Pathog 8: e1002502 doi:10.1371/journal.ppat.1002502 22291597 10.1371/journal.ppat.1002502 PMC3266935

[7] Kudchodkar SB, Del Prete GQ, Maguire TG, Alwine JC (2007) AMPK-mediated inhibition of mTOR kinase is circumvented during immediate-early times of human cytomegalovirus infection. J Virol 81: 3649–3651. 17215282 10.1128/JVI.02079-06 PMC1866081

[8] Spencer CM, Schafer XL, Moorman NJ, Munger J (2011) Human cytomegalovirus induces the activity and expression of acetyl-coenzyme A carboxylase, a fatty acid biosynthetic enzyme whose inhibition attenuates viral replication. J Virol 85: 5814–5824. 21471234 10.1128/JVI.02630-10 PMC3126312

[9] Moorman NJ, Cristea IM, Terhune SS, Rout MP, Chait BT, et al (2008) Human cytomegalovirus protein UL38 inhibits host cell stress responses by antagonizing the tuberous sclerosis protein complex. Cell Host Microbe 3: 253–262. 18407068 10.1016/j.chom.2008.03.002 PMC2759192

[10] Yu Y, Kudchodkar SB, Alwine JC (2005) Effects of simian virus 40 large and small tumor antigens on mammalian target of rapamycin signaling: small tumor antigen mediates hypophosphorylation of eIF4E-binding protein 1 late in infection. J Virol 79: 6882–6889. 15890927 10.1128/JVI.79.11.6882-6889.2005 PMC1112164

[11] Kumar SH, Rangarajan A (2009) Simian virus 40 small T antigen activates AMPK and triggers autophagy to protect cancer cells from nutrient deprivation. J Virol 83: 8565–8574. 19515765 10.1128/JVI.00603-09 PMC2738183

[12] Yu Y, Alwine JC (2006) 19S late mRNAs of simian virus 40 have an internal ribosome entry site upstream of the virion structural protein 3 coding sequence. J Virol 80: 6553–6558. 16775341 10.1128/JVI.00517-06 PMC1488956

[13] Gastaminza P, Cheng G, Wieland S, Zhong J, Liao W, et al (2008) Cellular determinants of hepatitis C virus assembly, maturation, degradation, and secretion. J Virol 82: 2120–2129. 18077707 10.1128/JVI.02053-07 PMC2258938

[14] Diamond DL, Syder AJ, Jacobs JM, Sorensen CM, Walters K-A, et al (2010) Temporal proteome and lipidome profiles reveal hepatitis C virus-associated reprogramming of hepatocellular metabolism and bioenergetics. PLoS Pathog 6: e1000719 doi:10.1371/journal.ppat.1000719 20062526 10.1371/journal.ppat.1000719 PMC2796172

[15] Mankouri J, Tedbury PR, Gretton S, Hughes ME, Griffin SD, et al (2010) Enhanced hepatitis C virus genome replication and lipid accumulation mediated by inhibition of AMP-activated protein kinase. Proc Natl Acad Sci U S A 107: 11549–11554. 20534540 10.1073/pnas.0912426107 PMC2895084

[16] Bose SK, Shrivastava S, Meyer K, Ray RB, Ray R (2012) Hepatitis C virus activates the mTOR/S6K1 signaling pathway in inhibiting IRS-1 function for insulin resistance. J Virol 86: 6315–6322. 22457523 10.1128/JVI.00050-12 PMC3372214

[17] Nakashima K, Takeuchi K, Chihara K, Hotta H, Sada K (2011) Inhibition of hepatitis C virus replication through adenosine monophosphate-activated protein kinase-dependent and -independent pathways. Microbiol Immunol 55: 774–782. 21895746 10.1111/j.1348-0421.2011.00382.x

[18] Kasai D, Adachi T, Deng L, Nagano-Fujii M, Sada K, et al (2009) HCV replication suppresses cellular glucose uptake through down-regulation of cell surface expression of glucose transporters. J Hepatol 50: 883–894. 19303158 10.1016/j.jhep.2008.12.029

[19] Daniel J, Maamar H, Deb C, Sirakova TD, Kolattukudy PE (2011) Mycobacterium tuberculosis uses host triacylglycerol to accumulate lipid droplets and acquires a dormancy-like phenotype in lipid-loaded macrophages. PLoS Pathog 7: e1002093 doi:10.1371/journal.ppat.1002093 21731490 10.1371/journal.ppat.1002093 PMC3121879

[20] Cocchiaro JL, Kumar Y, Fischer ER, Hackstadt T, Valdivia RH (2008) Cytoplasmic lipid droplets are translocated into the lumen of the Chlamydia trachomatis parasitophorous vacuole. Proc Natl Acad Sci U S A 105: 9379–9384. 18591669 10.1073/pnas.0712241105 PMC2453745

[21] Moser TS, Schieffer D, Cherry S (2012) AMP-activated kinase restricts Rift Valley fever virus infection by inhibiting fatty acid synthesis. PLoS Pathog 8: e1002661 doi:10.1371/journal.ppat.1002661 22532801 10.1371/journal.ppat.1002661 PMC3330235

[22] Zhang HS, Wu MR (2009) SIRT1 regulates Tat-induced HIV-1 transactivation through activating AMP-activated protein kinase. Virus Res 146: 51–57. 19720090 10.1016/j.virusres.2009.08.005

[23] Mehla R, Bivalkar-Mehla S, Zhang R, Handy I, Albrecht H, et al (2010) Bryostatin modulates latent HIV-1 infection via PKC and AMPK signaling but inhibits acute infection in a receptor independent manner. PLoS ONE 5: e11160 doi:10.1371/journal.pone.0011160 20585398 10.1371/journal.pone.0011160 PMC2886842

[24] Rosenzweig D, Smith D, Opperdoes F, Stern S, Olafson RW, et al (2008) Retooling Leishmania metabolism: from sand fly gut to human macrophage. FASEB J 22: 590–602. 17884972 10.1096/fj.07-9254com

[25] Rabhi I, Rabhi S, Ben-Othman R, Rasche A (2012) Sysco Consortium, (2012) et al Transcriptomic signature of Leishmania infected mice macrophages: a metabolic point of view. PLoS Negl Trop Dis 6: e1763 doi:10.1371/journal.pntd.0001763 22928052 10.1371/journal.pntd.0001763 PMC3424254

[26] Jaramillo M, Gomez MA, Larsson O, Shio MT, Topisirovic I, et al (2011) Leishmania repression of host translation through mTOR cleavage is required for parasite survival and infection. Cell Host Microbe 9: 331–341. 21501832 10.1016/j.chom.2011.03.008

[27] Takahashi M, Watari E, Shinya E, Shimizu T, Takahashi H (2007) Suppression of virus replication via down-modulation of mitochondrial short chain enoyl-CoA hydratase in human glioblastoma cells. Antiviral Res 75: 152–158. 17395278 10.1016/j.antiviral.2007.02.002

[28] Deretic V, Levine B (2009) Autophagy, immunity, and microbial adaptations. Cell Host Microbe 5: 527–549. 19527881 10.1016/j.chom.2009.05.016 PMC2720763

[29] Heaton NS, Randall G (2010) Dengue virus-induced autophagy regulates lipid metabolism. Cell Host Microbe 8: 422–432. 21075353 10.1016/j.chom.2010.10.006 PMC3026642

[30] Steele S, Brunton J, Ziehr B, Taft-Benz S, Moorman N, et al (2013) Francisella tularensis harvests nutrients derived via ATG5-independent autophagy to support intracellular growth. PLoS Pathog 9: e1003562 doi:10.1371/journal.ppat.1003562 23966861 10.1371/journal.ppat.1003562 PMC3744417

[31] Chong A, Wehrly TD, Child R, Hansen B, Hwang S, et al (2012) Cytosolic clearance of replication-deficient mutants reveals Francisella tularensis interactions with the autophagic pathway. Autophagy 8: 1342–1356. 22863802 10.4161/auto.20808 PMC3442881

